# The prevalence and immunological features of anti-glomerular basement membrane antibody in patients with HIV

**DOI:** 10.1186/s12882-020-02087-y

**Published:** 2020-10-08

**Authors:** Wen-jing Wang, Xiao-yu Jia, Zhao Cui, Yan Chen, Wei Wang, Jin-li Lou, Ming-hui Zhao, Sun Ying

**Affiliations:** 1 Department of Immunology, School of Basic Medical Sciences, Capital Medical University, Beijing, 100069 China; 2 Beijing Youan Hospital, Capital Medical University, Beijing, 100069 China; 3 Renal Division, Peking University First Hospital, Beijing, 100034 China; 4 Institute of Nephrology, Peking University, Beijing, 100034 China; 5 Key Laboratory of Renal Disease, Ministry of Health of China, Beijing, 100034 China; 6 Key Laboratory of CKD Prevention and Treatment, Ministry of Education of China, Beijing, 100034 China; 7 Peking-Tsinghua Center for Life Sciences, Beijing, 100871 China

**Keywords:** Anti-GBM antibodies, Antigen, IgG subclasses, HIV, AIDS

## Abstract

**Background:**

Anti-glomerular basement membrane disease (GBM) is an autoimmune disease caused by the deposition of circulating anti-GBM antibodies. Non-collagen region of α3 chain of type IV collagen (α3(IV)NC1) is one of the main target antigens, in which E_A_ and E_B_ are the most classical antigen epitopes. It has been reported that anti-GBM antibodies can be detected in HIV patients; however, its immunological characteristics are still unclear.

**Objectives:**

In this study, the positive rate of the anti-GBM antibodies in HIV and the immunological characteristics of the target antigens were clarified.

**Methods:**

A total of 93 HIV patients diagnosed in Beijing Youan Hospital from November 2017 to January 2018 were included. Enzyme-linked immunosorbent assay was used to measure the serum IgG autoantibodies specifically against GBM in these patients, as well as their subtypes and antigen spectra.

**Results:**

It was found that five out of the 93 patients with HIV had low to moderate levels of anti-GBM antibodies. However, these patients presented with no clinical manifestation of any kidney injury or pulmonary hemorrhages. Compared with HIV patients with negative antibodies, there were no significant differences in gender, age, CD4^+^T cell count and HIV viral load. All sera of five patients recognized non-collagenous domain1 (NC1) of alpha 3 chain of type IV collagen [(α3(IV)NC1] as classic anti-GBM patients, followed by α5(IV)NC1. The antibodies against α3(IV)NC1 were IgG3 predominant, while these antibodies did not react with either of the classic epitopes on α3 (E_A_ and E_B_).

**Conclusion:**

These data suggest a distinct immunological profile of anti-GBM antibodies in patients with HIV, and might explain the non-pathogenic features of HIV associated anti-GBM antibodies.

## Background

The acquired immunodeficiency syndrome (AIDS), first discovered in the 1980s, is an infectious disease caused by the human immunodeficiency virus (HIV). It can cause acquired immunodeficiency in humans, leading to various infections and malignant tumors, while end-stage patients may suffer from multiple organ dysfunction and death. Some studies have shown that 30% of patients may have abnormal renal functions, and those kidney damage in HIV-infected patients manifests in a variety of ways, including acute kidney injury (AKI), HIV-associated kidney disease, comorbid chronic kidney disease (CKD) and treatment-related kidney toxicity [1–3].

Many kinds of autoantibodies can be detected in HIV infected patients, including anti-nuclear (ANA),anti-neutrophil cytoplasmic (ANCA) and anti-glomerular basement membrane (GBM) antibodies [4].Anti-GBM antibodies are pathogenic of the anti-GBM disease which often manifests as the aggressive crescentic glomerulonephritis [3]. The target antigen of this antibody is the non-collagenous domain1 (NC1) of alpha 3 chain of type IV collagen (α3(IV)NC1), which normally expresses in the glomerular and alveolar basement membranes [1]. However, in most of the cases with anti-GBM antibodies detected in HIV patients, there were no obvious clinical manifestations of renal damage [4–6]. It is still in controversy whether these antibodies are pathogenic as the ones in classic anti-GBM patients. In the present study, therefore, we measured the anti-GBM antibodies in the serum of HIV patients and analyzed, for the first time to our best knowledge, the immunological characteristics of these antibodies in order to provide some clues.

## Methods

### Subjects and sera

Ninety-three patients with HIV, diagnosed in Beijing Youan Hospital from November 2017 to January 2018, were enrolled in the present study. The diagnosis of patients was based on the relevant provisions of WTO and China’s national industry standards [7]. According to the diagnostic criteria, all patients were diagnosed as the asymptomatic stage or AIDS since blood samples from patients with acute infection were difficult to collect. Patients with kidney injury caused by other causes, including recent severe trauma or surgery, and non-AIDS related acute and chronic infectious diseases, children, pregnant or lactating women were excluded. Sera of positive control were obtained from patients with classical anti-GBM disease diagnosed in Peking University First Hospital. Normal human sera were obtained from 20 healthy volunteers from Beijing Youan Hospital to establish the cutoff values.

All serum samples were collected and stored at − 80 °C until use. Clinical data of HIV patients were collected at the time of diagnosis and during follow-up. Siemens Advia2400 automatic biochemical analyzer was employed for renal function test, among which enzyme method and urease method were employed to measure creatinine and urea, respectively, while glomerular filtration rate was calculated by simplified MDRD formula. Sysmex XN series and relevant reagents were used for blood routine test (e.g. Hemoglobin), while Arkary AX4030 and necessary reagents were used for urine routine test (such as protein, red blood cell, etc.). BD FACS Canto II flow cytometer and the matching reagents were used for CD4 count, while Abbott real-time HIV-1 reagents were used for HIV viral load determination. These data were obtained from the patient’s medical records.

### Preparation of recombinant human a (IV) NC1 and chimeric proteins

The recombinant human α (IV)NC1 and chimeric protein were prepared as follows. Briefly, the cDNAs of the α1-α5(IV)NC1 were ligated into the X-type collagen triple helix guide sequence and cloned into the pcDNA plasmids, respectively. Then the plasmids were transfected into human embryonic kidney 293 cell line, while recombinant proteins were harvested from the culture solution after purification [8].

The constructs of E_A_ and E_B_ consist entirely of the α1(IV)NC1 domain, in which E_A_ contains 45 amino acids of α3(IV)NC1 E_A_ region, and E_B_ contains 37 amino acids of α3(IV)NC1 E_B_ region. Construction of non-E_AB_ was constructed in the context of α3(IV)NC1, in which the E_A_ and E_B_ regions were replaced by the corresponding amino acids of α1(IV)NC1 [8, 9] .

### Detection of anti-GBM antibodies in serum samples using enzyme-linked immunosorbent assay

A home-based enzyme-linked immunosorbent assay (ELISA) was performed to measure anti-GBM antibodies in the sera. In brief, a mixture of the recombinant human α1-α5(IV)NC1 proteins was diluted with carbonate buffer solution (CBS0.05 M, pH 9.6) to the concentration of 1.0 μg/mL for each antigen, and coated onto half of the wells of the polystyrene microtiter plate (Nunc, Roskilde, Denmark). The other half of the wells was coated with bovine serum albumin (BSA) as antigen-free wells, which was diluted at the same concentration in 0.05 M CBS to exclude non-specific binding. Incubation was carried at 37 °C for 60 min. After washing, the serum samples (1:100 diluted with a dilution buffer containing 0.64 M NaCl, 0.008 M Na_2_HPO_4_·12H_2_O, 0.003 M KCl, 0.002 M KH_2_PO_4_,0.1% Tween-20, pH 7.4) were then added to the antigen coated and antigen-free wells respectively. Incubation was performed again at 37 °C for 60 min. After washing, alkaline phosphatase–conjugated goat anti-human IgG (Fc specific, Sigma, USA, 1:6000) was added into the wells, and incubated againat37°C for 60 min. After washing, substrate solution (50 μL containing 1 mg/mL p-nitrophenyl phosphate, 1.0 M diethanolamine, 0.5 M MgCl_2_, pH 9.8, Sigma, USA) was added into the wells for color development which was measured spectrophotometrically at 405 nm (Bio-Rad, Tokyo, Japan) 15 min later. The net absorbance values were OD values of the antigen-coated wells minus the OD values of the antigen-free wells. Each serum sample was tested in duplication, while the samples were re-tested when the standard deviation between the wells > 10%. Serum samples from healthy volunteers were used to build up the cutoff values using mean + 2 SD for mixture α chains and the chimeric proteins.

### Detection of antigen and epitope specificity in sera of HIV patients with anti-GBM antibodies

To detect antigen and epitope specificity, plate wells were respectively pre-coated with each of the five-recombinant human α (IV)NC1 or the chimeric proteins containing E_A_, E_B_ and non-E_AB_(1 μg/mL per protein). Serum samples of HIV patients with anti-GBM antibodies were diluted at 1:50 and added in duplication into the wells at 37 °C for 60 min. After washing, alkaline phosphatase–conjugated goat anti-human IgG (Fc specific, Sigma, USA) was added into the wells and incubated at 37 °C for 60 min. Color was developed and measured as described above. Similarly, serum samples from healthy volunteers were used to build up the cutoff values using mean + 2 SD for each α chain and the chimeric protein.

### Detection of distribution of IgG subclass against α3(IV)NC1 in HIV patients with anti-GBM antibodies

The detection of IgG subclasses against α3(IV)NC1was performed as previously reported [8]. In brief, the recombinant human α3(IV)NC1 proteins were diluted at 1 μg/mL and coated onto half of the wells of a polystyrene microtiter plate. The other half of the wells was coated with BSA as antigen-free wells. Diluted serum samples at 1:100 were then added to the antigen and BSA coated wells respectively, and incubated at 37 °C for 60 min. After washing, horseradish peroxidase labeled mouse monoclonal antibodies against human IgG1, IgG2, IgG3, and IgG4 (Fc specific, Southern Biotech, USA, 1:2000) were added into the wells and incubated at 37 °C for 60 min. After washing, color was developed by adding 50 μL of substrate (Tetramethylbenzidine, TMB, Invitrogen, USA) into the wells for 15 min. Reaction was terminated by adding stop solution and color measured at450nm.Again, serum samples from healthy volunteers were used to build up the cutoff values using mean + 2 SD at the same steps.

#### Quality control

All laboratory test indexes were monitored by quality control materials with at least two levels. In the routine urine analysis, in case of positive alarm of red blood cells, white blood cells, proteins and nitrites, artificial microscopy was employed to eliminate the interference of false positive (such as mucous filaments).

#### Statistics

Continuous variables were expressed as means ± SD or medians. Comparison between continuous variables was conducted by *t* test for normally distributing data or nonparametric test for non-normally distributing data. Differences between qualitative data were analyzed using χ2 or Fisher exact test. All statistical analyses were two-tailed and *P* < 0.05 was considered significant different. Analysis was performed using the IBM SPSS statistics 24.0 software.

## Results

### The demographic and clinical data of patients with HIV

A consecutive of 93 patients diagnosed with HIV from 2017 to 2018 was enrolled in the present study, including 73 males and 20 females with an average age of 36.1 ± 10.7 years. Among these subjects, 48/93 (51.6%) patients were asymptomatic while the remaining were at the stage of AIDS. Thirty two (34.4%) of all the patients had a history of infections, including pulmonary, blood or other tissues, and 20 (21.5%) of them had laboratory evidence (supported by bacterial culture or molecular diagnosis).16 (17.2%) patients had urinary protein by semi quantitative analysis (1+ is equal to 0.3 g/L). All patients received anti-viral therapies and were followed up for a median time of 6 months. Two (2/93, 2.15%) patients died from pulmonary infection and multi-organ failure during their hospitalization, while 3(3/93, 3.23%) were lost at the end of follow-up. The demographic and clinical data are shown in Table 1.

**Table 1:** The Demographic and Clinical data of patients with HIV

Parameters	*n* = 93
Age (years)	36.1 ± 10.7
Gender (male/female)	73/20
Stage (Asymptomatic/AIDS)	45/48
CD4^+^T cell count (/μL) (median, range)	292 (0–1734)
HIV load (copies /mL) (median, range)	0 (0–3,257,132)
Infections (n, %)	20, 21.5%
Hemoglobin (g/L)	126.6 ± 30.8
Serum urea (mmol/L)	4.2 ± 1.2
Serum creatinine (μmol/L)	60.8 ± 16.7
eGFR (ml/min/1.73m^2^)	120.8 ± 20.3
Positive urinary protein (*n*, %)	16, 17.2%

### The prevalence of circulating anti-GBM antibodies in patients with HIV

Serum samples of 20 normal controls were collected and used to build up the cutoff values of anti-GBM antibodies. The mean OD value of healthy controls was 0.050 ± 0.110, and cutoff value was 0.270. A total of 5 sera (5.37%) from the 93 patients showed reactivity against a mixture of all five human α (IV)NC1 chains. Among them, 4 were male and one female, with a mean age of 39.8 ± 11.3 years.

None of these 5 patients with anti-GBM antibodies had hemoptysis or any kind of kidney injuries including urinary protein or hematuria. However, these 5 patients presented significantly higher levels of serum urea (5.3 ± 0.6 vs. 4.2 ± 1.2 mmol/L, *p* = 0.027), creatinine (78.5 ± 25.1 vs. 59.7 ± 15.8 μmol/L, *p* = 0.014) and lower levels of estimated glomerular filtration rate (eGFR, 103.2 ± 24.1 vs. 121.7 ± 19.9 ml/min/1.73m^2^, *p* = 0.048), compared with the HIV patients without the antibodies. In HIV patients without anti-GBM antibodies, urinalysis indicated urinary protein (mostly 1+) in 16 cases (18.8%,16/88) and hematuria (mostly 1+) in 3 cases (3.41%,3/88), respectively.

There were no significant differences in HIV viral load and CD4^+^ T lymphocyte count between HIV patients with or without anti-GBM antibodies (Table 2). No matter either CD4^+^ T cell count less than 200/μL or 400/μL was used as a marker of immune deficiency, there were no differences in the renal function and the prevalence of the anti-GBM antibodies between groups (Table 3).

**Table 2:** Comparison of clinical features of HIV patients with and without anti-GBM antibodies

Parameters	with anti-GBM antibodies (*n* = 5)	without anti-GBM antibodies (*n* = 88)	*P* value
Age (years)	36.6 ± 9.8	36.3 ± 11.0	0.939
Gender (male/female)	4/1	69/19	0.933
Stage (Asymptomatic/AIDS)	4/1	44/44	0.192
CD4^+^T cell count (/μL)	520.6 ± 171.6	146.8 ± 214.0	0.308
CD4/CD8 ratio	0.77 ± 0.62	0.07 ± 0.05	0.235
HIV Load (Detected/NOT)	1/4	29/59	0.052
Antiviral drug (Lamivudine/ Tenofovir fumarate/ Eferene)	5/4/5	64/53/46	0.873
Hemoglobin (g/L)	141.0 ± 32.1	125.8 ± 30.7	0.284
Serum urea (mmol/L)	5.3 ± 0.6	4.2 ± 1.2	0.027*
Serum creatinine (μmol/L)	78.5 ± 25.1	59.8 ± 15.7	0.014*
eGFR (ml/min/1.73m^2^)	103.2 ± 24.1	121.8 ± 19.8	0.046*
Infections (*n*, %)	3, 60%	17, 19.3%	0.031*
Positive urinary protein (*n*, %)	0	16, 18.2%	0.295

*P < 0.05

**Table 3:** Comparison of anti-GBM antibody level and renal function in CD4 count

CD4^+^ T cell count (/μL)	< 200 (*N* = 35)	> 200(*N* = 58)	*P*	< 400 (*N* = 56)	> 400(*N* = 37)	*P*
N_GBM(+)_	1	4	0.34	1	4	0.34
OD_GBM level_	0.06 ± 0.07	0.03 ± 0.09	0.06	0.05 ± 0.07	0.03 ± 0.10	0.54
Serum urea (mmol/L)	4.2 ± 1.0	4.3 ± 1.2	0.71	4.3 ± 1.2	4.2 ± 1.2	0.67
Serum creatinine (μmol/L)	62.0 ± 20.0	60.0 ± 14.5	0.57	60.2 ± 17.7	61.6 ± 15.2	0.69
eGFR (ml/min/1.73m^2^)	121.9 ± 29.6	120 ± 11.9	0.68	122.2 ± 24.7	118.6 ± 10.5	0.40

*P < 0.05

However, the prevalence of infections in patients with anti-GBM antibodies seemed to be higher. Three out of the 5 patients (60%, 3/5) possessing anti-GBM antibodies had infections, compared to the corresponding figure of 17/88 (19.3%) in anti-GBM negative patients (*P* = 0.031).

All patients involved in the study had received free antiviral treatment (used Lamivudine/Zidolamivudine, Tenofovir fumarate, Lopinavir, Eferene or Latiravir), including the patients with anti-GBM antibodies (treated with Lamivudine, Tenofovir fumarate or Eferene). There were no differences in urine protein, serum creatinine, urea and eGFR among the three drugs.

### Antigens and epitope specificity of anti-GBM antibodies in the sera of HIV patients

The recombinant human α1 to α5(IV)NC1 were used to determine antigen specificity of anti-GBM antibodies. Among these 5 patients, one failed to react with either of the five α chains. Sera of the remaining four patients (4/4, 100%) all recognized α3(IV)NC1, while 2 sera (2/2, 50%) recognized α5(IV)NC1. Only one patient (1/5, 20%) reacted with α1(IV)NC1, α2(IV)NC1, α4(IV)NC1 (Table 4). Sera from positive controls recognized both E_A_ and E_B_. However, none of the three chimeric proteins was recognized by the sera from the 4 HIV patients with anti-α3(IV)NC1 antibodies (Table 4).

**Table 4:** Target antigens of circulating anti-GBM antibodies in HIV patients

	Antigen distribution					Epitope on α3(IV)NC1		
	α1(IV)NC1	α2(IV)NC1	α3(IV)NC1	α4(IV)NC1	α5(IV)NC1	E_A_	E_B_	Non-E_AB_
P1	0.050	−0.051	− 0.024	− 0.04	− 0.043	–	–	–
P2	**0.155**^**△**^	**0.612**^**△**^	**0.324**^**△**^	**0.079**^**△**^	**0.095**^**△**^	0.026	−0.016	0.028
P3	0.039	0.031	**0.142**^**△**^	−0.086	**0.131**^**△**^	−0.006	0.009	0.011
P4	0.001	0.015	**0.138**^**△**^	−0.001	0.024	0.005	0.066	0.151
P5	0.031	−0.003	**0.132**^**△**^	0.005	0.011	0.043	0.037	0.067
Normal controls	0.017 ± 0.034	0.037 ± 0.070	0.012 ± 0.027	−0.036 ± 0.051	0.009 ± 0.028	0.052 ± 0.049	0.036 + 0.060	0.078 ± 0.064
Cut-off value	0.085	0.178	0.066	0.067	0.066	0.150	0.156	0.205

^**△**^Positive OD value

### IgG subclass distribution of serum antibodies against α3(IV)NC1

IgG subclasses were further detected in the 4 HIV patients with anti-α3(IV)NC1antibodies. Among them, IgG3 was the dominant subtype (100%, 4/4), while IgG1 and IgG4 were detected in 1/4 case, respectively. IgG2 subclass was not detected in any of sera (Table 5).

**Table 5:** IgG subclass distribution of circulating antibodies against α3(IV)NC1 in HIV patients

	IgG subclass distribution			
	IgG1	IgG2	IgG3	IgG4
P1	–	–	–	–
P2	−0.07	−0.002	**0.196**^**△**^	0.004
P3	0.078	0.005	**0.138**^**△**^	−0.015
P4	0.133	−0.003	**0.252**^**△**^	**0.281**^**△**^
P5	**0.225**^**△**^	0.007	**0.136**^**△**^	−0.137
Normal controls	0.067 ± 0.048	−0.003 ± 0.017	−0.010 ± 0.029	− 0.010 ± 0.054
Cutoff value	0.163	0.031	0.048	0.098

^**△**^Positive OD value

### Follow up of HIV patients with anti-GBM antibodies

All the 5 patients possessing anti-GBM antibodies were followed up for 6–12 months (Table 6). Among them, 2 patients showed slight decline of eGFR (decreased by 10.4 and 11.4% respectively). One patient, whose serum recognized all five α chains, developed elevated level of urinary protein (semi-quantitative detection at 1.0 g/L). The remaining 3 patients showed no further renal impairment.

**Table 6:** Clinical data of anti-GBM-positive HIV patients during follow-up

No	Gender	Stage	on the time of testing						follow-up after one year					
			CD4	Viral load	UPRO	UREA	CRE	eGFR	CD4	Viral load	UPRO	UREA	CRE	eGFR
			(/μL)	(copy/ml)		(mmol/L)	(μmol/L)	(ml/min/1.73m^2^)	(/μL)	(copy/ml)		(mmol/L)	(μmol/L)	(ml/min/1.73m^2^)
P1	Male	AIDS stage	42	TND	–	6.4	109.3	69.1	58	TND	–	4.2	84.8	86.9
P2	Male	Asymptomatic	407	791,856	–	5.4	74.0	124.5	451	42	2+	5.0	84.5	111.6
P3	Male	Asymptomatic	440	< 40	–	4.9	67.1	119.1	460	TND	–	6.2	73.1	114.2
P4	Male	Asymptomatic	776	TND	–	5.2	96.5	86.7	608	TND	–	5.2	106.9	76.8
P5	Female	Asymptomatic	459	TND	–	5.0	45.4	116.7	645	TND	–	5.6	51.9	110.8

## Discussion

Kidney dysfunction caused by HIV infection is related to a number of reasons including the course of disease, viral load status, drug treatments, co-infections, tumorigenesis and metabolic disorders [10, 11]. It has been reported that HIV patients have autoantibodies against GBM, complicating the dissection of the causes for kidney involvement [4]. Although classic anti-GBM antibodies are recognized to be pathogenic, most HIV cases with anti-GBM antibodies manifest no features of anti-GBM nephritis [4, 5]. In order to further elucidate the pathogenesis of these antibodies, we investigated, for the first time to our best knowledge, the immunological characteristics of the anti-GBM antibodies in HIV patients and their associations with clinical data.

A total of 5 out of 93 HIV patients possessed low to moderate levels of anti-GBM antibodies. However, no kidney injury or hemoptysis was found in the 5 patients at the time of testing. Therefore, these antibodies in HIV patients seem to be non-pathogenic, as consistent with previous reports [5, 6]. Because there were no differences in the concentrations of serum creatinine of patients with any of the antiviral drug treatment, this suggests that these antiviral drugs did not cause renal damage in these patients yet, at least at the time of the study. It has been reported that the production of anti-GBM antibody is related to CD4^+^ T cell count, in which there is a significant correlation between the presence of anti-GBM antibodies and CD4 counts less than 400/μL [4]. However, we didn’t find such tendency, even though CD4 count less than 200/μL was used as a marker of severe immunodeficiency. In this case, therefore, the relationships between CD4^+^ T cell count and autoantibody production in HIV patients still need to be further clarified.

Among the patients, one patient failed to react with any of the five-alpha chain, which is probably due to the low reactivity of the antibodies to each alpha chain, though the total IgG against all the five alpha chains could be detected. In the present study, one of the 5 anti-GBM positive patients, who had the antibodies against all 5 α chains, also had a high viral load, suggesting that the CD4^+^ T cell count might associate with HIV viral load in the point of view of antigenic reorganization. However, there was no significant difference in CD4^+^ T cell count and HIV viral load between HIV patients with or without the antibodies, probably because all patients in our study had taken antiviral drugs. Furthermore, our data revealed that the prevalence of anti-GBM antibodies was associated with infections. Previous studies have shown that *P. carinii* alveolar injury or the host response to the organism might affect the Goodpasture antigen or a similar antigen, because Goodpasture’s syndrome could be triggered by an alveolar lesion induced by *P.carinii* pneumonia [5, 12]. Anti-GBM disease associated with other infections including dengue virus has also been reported [13]. Taken together, these suggest that there might be an important link between infection and the emergency of anti-GBM antibodies, which needs to be further investigated.

Although the production of autoantibodies associated with HIV infections is thought to be caused by poly-clonal activation of CD4^+^T cells, we found that anti-GBM antibodies in HIV patients were rather specific and recognized a relatively narrower antigen spectrum. Almost all sera recognized α3(IV)NC1 and half of them reacted with α5(IV)NC1, while the recognition of other α chains was limited. It has been shown that the main target antigen recognized by GBM antibody is α3(IV)NC1, and that the level of α3(IV)NC1 antibody is a key factor in the extent of kidney damage in classic anti-GBM diseases [14, 15]. Recently, antibodies against α5(IV)NC1 have been proved to be pathogenic in Goodpasture’s disease as well [16]. However, in the present study, none of these HIV patients with anti-GBM antibodies had clinical evidence of renal injuries. It is worth noting that most of the anti-GBM antibodies in these patients were borderline positive, with only one patient possessing moderately elevated level of antibodies against α3(IV)NC1. Moreover, none of these sera recognized the two major epitopes on α3(IV)NC1, namely E_A_ and E_B_. It has been shown that patients with high levels of circulating antibodies against the specific epitopes E_A_ and E_B_ have a more severe renal disease at diagnosis as well as a worse prognosis [17]. Data derived from animal models suggest a non-pathogenic but augmenting effect of anti-EB antibodies in the development of crescentic glomerulonephritis [18]. Some other studies have demonstrated that the levels of anti-E_B_ antibodies in human anti-GBM disease are positively associated with severity of renal damage [17]. Therefore, it could be speculated that the antibodies in these patients might recognize non-pathogenic epitopes on α3 and α5(IV)NC1, therefore exerting no kidney injuries.

Savige et al. have shown that plasma samples from 18 of 105 HIV infected individuals are positive for anti-GBM antibodies [4]. Most of the patients also have borderline levels of anti-GBM antibodies, though the prevalence is much higher than those involved in the present study. The authors suggest that these antibodies may arise from polyclonal activation, or may be due to “sticky” serum [4], especially when sera diluted at 1:8. In the present study, all serum samples were diluted at 1:100 to reduce false positivity, which might be one of the reasons for a lower prevalence of anti-GBM antibodies in our study.

### Limitations

There are certain limitations in the present study. For example, the sample size is small, while the research time is still short, and this is a single center study. Furthermore, it is difficult to determine the specific time of HIV infection for each individual patient. In fact, many patients were only found in the occasional physical examination, and some patients went to the hospital for examination when they had other clinical symptoms. In this case, it is hard to know the exact time when the patients were actually infected with HIV virus. In addition, HIV viral load and CD4^+^T count might affect the GBM antibody production. The patients included in the present study had received different anti-virus drugs and treatments, which may interfere with the results. At last, the epitope peptide corresponding to the positive sample has not been found, which needs further investigation.

## Conclusions

Five out of 93 HIV patients were found to have low to moderate levels of anti-GBM antibodies. However, none of the patients manifested any kind of kidney injuries or pulmonary hemorrhages. These antibodies recognized a specific and narrow antigen spectrum encoding α3 and α5(IV)NC1, but had a distinct epitope repertoire. The differences in immunological characteristics may explain the non-pathogenic features of HIV associated anti-GBM antibodies.

## Data Availability

Our database contains sensitive data, which can directly provide clinical data and personnel information about our patients and lead to the identification of these patients. Therefore, according to the restrictions and provisions of the relevant organizations, these data cannot be publicly provided. However, if the requirements are reasonable, datasets used and/or analyzed in this study can be obtained from the authors.
